# Innovations in conservative and surgical therapy for Ménière’s disease—narrative review

**DOI:** 10.3389/fneur.2026.1866969

**Published:** 2026-07-28

**Authors:** Agnieszka Jasińska-Nowacka, Damian Zienkiewicz, Maria Makuszewska, Tomasz Wojciechowski, Michał Kaczmarczyk, Kazimierz Niemczyk

**Affiliations:** 1 Department of Otorhinolaryngology, Head and Neck Surgery, Medical University of Warsaw, Warsaw, Poland; 2 Students’ Scientific Group, Department of Otorhinolaryngology, Head and Neck Surgery, Medical University of Warsaw, Warsaw, Poland; 3 Department of Descriptive and Clinical Anatomy, Medical University of Warsaw, Warsaw, Poland; 4 Department of Otolaryngology and Oncological Laryngology with Cranio-Maxillofacial Surgery Unit, Military Institute of Medicine—National Research Institute, Warsaw, Poland

**Keywords:** betahistine, canal plugging, ebselen, endolymphatic duct blockage, intratympanic steroids, Ménière’s disease, vestibular neurectomy

## Abstract

Ménière’s disease (MD) is an inner ear disorder of poorly understood etiology, and its management remains symptomatic. This review presents novel therapeutic approaches to control vertigo, improve audiological function, and enhance patient outcomes in MD. The literature was reviewed to explore conservative and surgical methods under current investigation. Recent advances in pharmacological therapy target oxidative stress, immune-mediated pathways, and neuromodulatory mechanisms. Clinical trial reports evaluating the efficacy of new oral treatment-ebselen were included. Preliminary report describing treating patients with MD and migaine with rimegepant was analyzed. Novel strategies to improve betahistine bioavailability were also reviewed. Authors reported modifications to intratympanic steroid therapy with OTO-104 and SPT-2101 (sustained-release intratympanic dexamethasone hydrogel). Contemporary surgical approaches increasingly aim to preserve hearing and, when feasible, partial vestibular function. Among surgical techniques, endolymphatic sac surgery with various modifications, endolymphatic duct blockage, canal plugging, and transcanal neurectomy were analyzed. Advances in pharmacological and surgical strategies offer new options for MD symptom control, but their effectiveness remains to be confirmed in larger, long-term trials. At the same time, these innovative therapeutic approaches provide valuable insights into the underlying pathophysiological mechanisms of the disease and may stimulate further research into its etiology. A better understanding of disease etiology is essential for developing causal treatments.

## Introduction

Ménière’s disease (MD) is characterized by attacks of vertigo accompanied by sensorineural hearing loss, tinnitus, and/or aural fullness (1, 2). The diagnosis of Ménière’s disease remains challenging because no gold-standard diagnostic test is currently available. Consequently, diagnosis is based primarily on clinical criteria established by international consensus guidelines, which classify the disease as either definite or probable (1–5). Definite Ménière’s disease is diagnosed when the following criteria are met: (1) two or more spontaneous episodes of vertigo lasting 20 min to 12 h; (2) audiometrically documented low- to mid-frequency sensorineural hearing loss in the affected ear, recorded before, during, or after an episode of vertigo; (3) fluctuating aural symptoms, including hearing loss, tinnitus, or aural fullness, affecting the involved ear; and (4) symptoms not better explained by another vestibular disorder. Given the poorly understood etiology of MD, management remains symptomatic. According to the latest guidelines established by the American Academy of Otolaryngology-Head and Neck Surgery (AAO-HNS) (1) and by Japanese and European societies (3–5), MD therapy is based on an escalation protocol. Initial management includes dietary modifications such as low-sodium intake, adequate water intake, and restriction of alcohol and caffeine (6–8). However, the evidence supporting these recommendations is limited and mainly from observational studies. Beyond vestibular symptoms, Ménière’s disease substantially impairs quality of life and is frequently associated with anxiety and depressive symptoms, emphasizing the need for effective symptom control and comprehensive patient management (9). Non-pharmacological management includes lifestyle modifications such as stress reduction, improved sleep, and psychotherapy for patients with mood disorders and high anxiety (1, 3–5). Although there are some reports describing clinical improvement, according to Cochrane review there is currently no high-quality evidence supporting the effectiveness of commonly recommended lifestyle and dietary interventions as the available evidence is based on only two small randomized trials (10).

Acute management of MD focuses on alleviating symptoms during attacks; vertigo and nausea can be treated with antihistamines, benzodiazepines, anticholinergics, antidopaminergics, and 5-HT3 antagonists. Long-term management aims to reduce the frequency and severity of vertigo episodes and preserve hearing. In Europe and Japan, first-line oral therapy for MD is betahistine at 48–96 mg/day, depending on the country (3–5); this drug is not used in the USA as it is not FDA-approved. For attack prevention, diuretics such as hydrochlorothiazide, acetazolamide, and loop diuretics may be used as second-line therapy. Intratympanic therapies, including steroids and gentamicin, are also proven effective (5). About 10% of patients require surgical intervention for satisfactory vertigo control and improved quality of life (1, 4, 5). Among invasive procedures, vestibular-preserving and destructive techniques can be offered.

The relationship between cochlear and vestibular dysfunction in Ménière’s disease appears to be complex, as auditory and vestibular symptoms may progress independently (11, 12). This functional dissociation further supports the heterogeneous nature of the disease and underscores the need for individualized diagnostic and therapeutic strategies.

Notably, recent Cochrane systematic reviews consistently demonstrate that the quality of evidence supporting MD treatment including lifestyle modifications, oral pharmacotherapy, intratympanic corticosteroids and gentamicin, positive pressure therapy, and surgical procedures remains low or very low (13–17). Thus, adequately powered randomized controlled trials with standardized diagnostic criteria and outcome measures are necessary to establish evidence-based recommendations. Taking into consideration limited number of large randomized controlled trials in MD, in this review we included studies investigating innovative therapeutic approaches. Although many of these interventions are supported only by preliminary reports, they provide valuable insights into potential future treatment strategies.

A variety of conservative and surgical treatment methods are being investigated to improve vertigo control and achieve a satisfactory functional level in patients with MD. In the present study, a literature review on novel therapeutic approaches was presented. The review focused on the effectiveness of each method in reducing vertigo, as this is considered the main goal of MD therapy. Still, we aimed to discuss the audiological outcomes and changes in patients’ quality of life, and to comprehensively evaluate treatment outcomes.

## Methodology

A non-systematic search of PubMed, Scopus, and Web of Science was performed to identify relevant studies published up to March 2026. Search terms included “Ménière’s disease,” “endolymphatic hydrops,” “vertigo,” “betahistine,” “ebselen,” “SPI-1005,” “OTO-104,” “SPT-2101,” “endolymphatic sac surgery,” “endolymphatic duct blockage,” “canal plugging,” and “vestibular neurectomy.” The literature search for innovative MD therapies was limited to English-language publications. However, a small number of non-English articles were additionally cited when they represented provided important historical and anatomical context that could not be obtained from equivalent English-language publications. As this is a narrative review, no formal systematic review methodology was performed.

Priority was given to randomized controlled trials and meta-analyses. Still, preliminary clinical papers were identified through manual screening of the bibliographies of selected articles. To provide a comprehensive overview of emerging therapeutic strategies, this review also includes selected data from conference presentations and sponsor communications when no peer-reviewed full-text publications were available. The final selection of publications was based on their relevance to innovative pharmacological and surgical treatment strategies for Ménière’s disease.

## Oral pharmacotherapy

**Figure 1:**
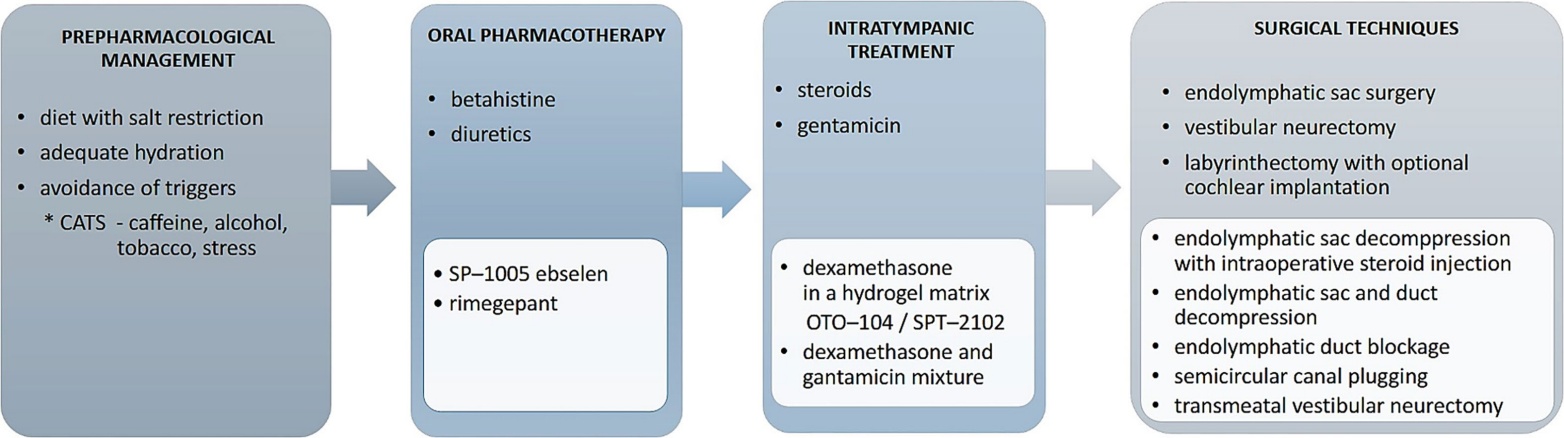
Escalation treatment algorithm for Ménière’s disease, including established therapeutic strategies and innovative treatment methods. Investigational pharmacological agents and surgical techniques are grouped according to the corresponding treatment categories.

As the etiology of MD remains poorly understood, there is currently no approved causal pharmacotherapy for MD (1, 3–5). However, novel therapeutics for inner ear disorders are being developed (Table 1; Figure 1). Most of them focus on an otoprotective approach. Drugs for regenerative approaches are also under development at early stages (preclinical, phase 1/2) (18).

**Table 1:** Emerging conservative therapies in Ménière’s disease.

Administration route	Substance	Mechanism of action
Oral	Ebselen (SPI-1005)	Glutathione peroxidase mimic—antioxidant. Reduces oxidative stress and inflammation via Nrf2 pathway.
	Rimegepant	CGRP receptor antagonist. Reduces neurogenic inflammation. Modulates migraine-related vestibular pathways.
Intratympanic	OTO-104	Sustained-release corticosteroid. Local anti-inflammatory effect in the inner ear.
	SPT-2101	Targeted, prolonged dexamethasone delivery. Sustained anti-inflammatory action.

*Mechanisms are simplified and based on current understanding of pharmacological targets relevant to inner ear disorders and vestibular dysfunction. CGRP, calcitonin gene-related peptide; Nrf2, Nuclear factor erythroid 2-related factor 2.

A recent Cochrane systematic review identified only 10 randomized controlled trials (total population of 848 patients) evaluating systemic pharmacotherapy including betahistine, diuretics, antivirals, and corticosteroids (13). Across all methods, the certainty of evidence was rated as low or very low because of small sample sizes, methodological limitations, heterogeneous outcome measures, and inconsistent follow-up periods.

### Betahistine (AM-125)

Betahistine remains the first-line pharmacological treatment for Ménière’s disease in many European and Asian countries, however it is not approved by the FDA (1–3, 5). In a 12-year retrospective study involving 105 patients with definite Ménière’s disease, betahistine treatment was associated with a significant reduction in the frequency and duration of vertigo episodes (*p* < 0.00001 and *p* = 0.000098, respectively) (19). The mean daily dose was 87.5 ± 27.2 mg, and no significant association was found between higher doses and treatment efficacy, supporting an individualized dosing strategy. Combination therapy with piracetam was associated with a further reduction in vertigo attacks, although these findings require confirmation in prospective studies.

Despite its widespread use, its clinical efficacy remains the subject of ongoing debate. One of the major betahistine limitations is poor oral bioavailability resulting from extensive first-pass metabolism. Orally administered betahistine is metabolized by monoamine oxidase-B (MAO-B), resulting in extremely low bioavailability and limiting its therapeutic potential.

Intranasal betahistine (AM-125) has been developed to improve bioavailability and provide more rapid systemic absorption. In a phase 2 randomized placebo-controlled trial involving 124 patients with surgery-induced acute vestibular syndrome (vestibular schwannoma resection, labyrinthectomy or vestibular neurectomy), AM-125 demonstrated a favorable safety profile and showed a trend toward faster vestibular compensation. Authors described greater improvement in tandem Romberg performance, although the difference did not reach conventional statistical significance (*p* = 0.08). Patients treated with AM-125 had higher rate of spontaneous nystagmus resolution, and improved patient-reported vestibular outcomes compared with placebo. Results of the Vestibular Rehabilitation Benefit Questionnaire (VRBQ) analyzing self-assessed dizziness, showed a trend favoring AM-125 group (1.2–3.8 percentage points). Although these findings were obtained in patients with iatrogenic postoperative acute vestibular injury rather than conservative treatment of Ménière’s disease, they support further investigation of alternative betahistine formulations in vestibular disorders (20).

Another strategy to improve betahistine bioavailability is inhibition of its metabolism by monoamine oxidase-B (MAO-B). In a phase 1 pharmacokinetic study involving 15 healthy volunteers, Strupp et al. (21) reported preliminary findings that co-administration of the MAO-B inhibitor selegiline (5 mg/day) increases betahistine bioavailability by a factor of about 80–100 (*p* < 0.0001), without raising safety concerns. In the study, higher systemic exposure was achieved, suggesting that MAO-B inhibition may be one of the principal pharmacokinetic limitations of betahistine. Whether this translates into improved clinical efficacy in patients with Ménière’s disease remains to be confirmed in randomized placebo-controlled trials.

### Ebselen (SPI-1005)

Recently, studies on the anti-inflammatory drug ebselen (SPI-1005) in MD treatment were published (22–25). SPI-1005 acts as a glutathione peroxidase mimic and free radical scavenger, protecting cellular components from oxidative damage. These properties led to trials on its use in MD as an otoprotective drug. In preclinical models, SPI-1005 mimicked the cochlear main antioxidant enzyme, protecting spiral ganglion neurons and hair cells, and induced cytoprotective genes by activating the Keap1-Nrf2 pathway. It also reduced noise-induced endolymphatic hydrops in animals, suggesting its relevance for treating idiopathic hydrops in humans (22). Early-phase randomized clinical trials have demonstrated that ebselen is generally safe and well-tolerated, with no serious treatment-related adverse events or deaths reported during treatment periods of up to 28 days (24). In a phase 1b study, ebselen showed clinically relevant improvements in tinnitus loudness (measured by VAS) compared to placebo (25). However, the same study found no significant difference in overall Vertigo Symptoms Scale (VSS) and Tinnitus Functional Index (TFI) scores between ebselen and placebo groups (22, 26). The VSS and TFI assess vertigo-related symptoms and tinnitus impact on daily life, respectively.

In a phase 2b multicenter study, efficacy for Meniere’s disease was evaluated, using pure-tone audiometry (PTA) and the Words-in-Noise Test (WIN), which measures monosyllabic word recognition in background noise. The intent-to-treat analysis included 124 patients who received at least one dose of the study medication. According to a preliminary sponsor report, the group treated with 400 mg doses of SPI-1005 showed a 76% relative improvement (60% vs. 34% placebo) in WINT scores and a 95% relative improvement (39% vs. 20% placebo) in PTA scores (24). Subsequently, on September 17, 2019, the FDA designated SPI-1005 as a Fast Track development program for Meniere’s disease.

The first phase 3 study of SPI-1005 for MD (STOPMD-3, ID: NCT04677972) was completed on July 25, 2024. Positive results were announced on December 10, 2024, on the sponsor’s website (23) and at the 48th Annual MidWinter Meeting of the Association for Research in Otolaryngology (24). Two hundred twenty-one adult subjects with a history of definite MD and active symptoms received 28 days of either SPI-1005 or placebo. Hearing and vestibular function were reassessed monthly through 84 days. The SPI-1005 group showed significant PTA improvement (57.9% vs. 36.5% for placebo, *p* = 0.0037) and greater WIN improvement (42.1% vs. 27.1% for placebo, *p* = 0.0336) on day 84 (23, 24). The report and conference abstract do not mention vestibular function results on day 84. Compliant patients could join an open-label extension for up to 12 months, starting after the initial phase. Auditory and vestibular function were checked every 3 months. The sponsor claims responder rates for PTA and WIN continued to improve, and patient-reported measures of tinnitus, vertigo, aural fullness, and dizziness all improved from baseline (≥30% on average, *p* < 0.001), but gives no further details (24). Although the relative improvements in WINT and PTA outcomes are encouraging, critical interpretation of these findings is necessary until complete data become available in the peer-reviewed literature.

Nowadays, in the USA, ebselen is the only orally administered investigational drug that has progressed to phase 3 testing for hearing loss, highlighting its significance in this field. Additionally, ebselen is being studied as a potential treatment for treatment-resistant depression (OxIMP, ID: NCT05117710) and for moderate and severe COVID-19 (IDs: NCT04484025, NCT04483973).

### Rimegepant

In the last decade, a small-molecule calcitonin gene-related peptide (CGRP) antagonist, rimegepant, was developed as a potentially antimigraine drug. In 2023, it was approved by the EMA and is now available for the treatment of migraine in Europe. CGRP modulates vasodilation, pain signaling, and immunomodulation, particularly in the nervous system (27). The relationship between MD and migraine has been described in several studies, suggesting a common pathophysiology. Migraine headaches are five times more common in MD patients than in the general population. Moreover, 95% of MD patients complain of migraine features including sensitivity to visual input, head motion, light, sound, smells, and weather changes (28). Interestingly, transient endolymphatic hydrops has been visualized using delayed gadolinium-enhanced MRI of the inner ear following an episode of migraine headache (29).

Lately, a pilot study regarding the treatment of coexisting MD and migraine was conducted in Switzerland (30). Six patients with definite MD and comorbid migraine were enrolled in the study and treated with rimegepant, an oral calcitonin gene-related peptide (CGRP) receptor antagonist. The treatment was effective in relieving migraine headaches, furthermore, all patients have been free of vertigo attacks since the first dose during a follow-up ranging from 5 to 31 months. It is worth noting that two patients took their first pill at the beginning of the MD episode and symptoms resolved within 1 h–1.5 h. Preliminary clinical results should not be regarded as conclusive evidence. Instead, this case series may be viewed as generating a hypothesis and highlighting a potentially novel avenue for future research into the pathophysiology and treatment of Ménière’s disease.

Chu et al. (31) conducted the first prospective study of rimegepant 75 mg specifically for the acute treatment of vestibular symptoms. Eighteen patients with vestibular migraine or probable vestibular migraine rated five core symptoms at baseline and at multiple time points post-treatment. All symptom scores showed a decreasing trend starting at 30 min, with statistically significant reductions for all symptoms except headache by 2 h. By 24 h, all patients achieved recovery from moderate/severe symptoms to absent/mild for all five symptom domains. No adverse events were reported. While these preliminary results are promising, both studies are limited by small sample sizes and lack of placebo control. The natural history of VM and MD includes spontaneous symptom resolution, and placebo responses in vestibular disorders are known to be substantial. Nonetheless, the consistent signal across two independent studies, one in MD with migraine and one in VM, suggests that CGRP antagonism may represent a novel therapeutic mechanism for vertigo control. Further randomized controlled trials are warranted.

## Intratympanic treatment

The next step in MD therapy involves local drug administration into the tympanic cavity (Table 1; Figure 1). Depending on the patient’s disease course and hearing level, corticosteroids or aminoglycosides may be indicated (1, 3–5, 32). A limitation of conventional intratympanic therapy is the need for repeated drug injections.

Corticosteroids have been shown to modify inner ear epithelial ion transport by affecting sodium channels and sodium-potassium ATPases. The outflow of sodium cations from the labyrinth is believed to reduce endolymph volume, thereby alleviating MD symptoms. Repeated intratympanic injections of aqueous corticosteroid solutions have already been associated with clinical improvement in patients with MD. Administration directly into the middle ear allows maximal exposure of the inner ear to the drug, minimizing the risk of systemic adverse effects (32).

A recent Cochrane systematic review evaluating intratympanic corticosteroids for MD identified only 10 randomized controlled trials (total population of 952 patients) (14). Authors evaluated the certainty of evidence as low or very low due to small sample sizes, methodological limitations, and concerns regarding publication bias as some results were not published yet. Review analyzing gentamicin included only five small randomized or quasi-randomized studies (total population of 137 patients), thus very low certainty of evidence was concluded by the authors of the review (15). However, most randomized controlled trials included in these Cochrane reviews were published more than a decade ago, before the introduction of current MRI endolymphatic hydrops visualization and updated diagnostic criteria. Consequently, there is a need to analyze recently developed treatment protocols.

### OTO-104

OTO-104 is a novel drug that is a suspension of dexamethasone in a buffered solution of the thermoreversible glycol polymer, poloxamer 407 (33). The triblock poloxamer class of biopolymers is appealing due to its mucoadhesive properties and a safety profile in humans. When injected intratympanically, poloxamer suspension converts from a liquid to a hydrogel matrix that provides sustained release of dexamethasone within the inner ear for at least 10 days in humans and 2–3 months in animals (33, 34). Due to all those properties, OTO-104 was proposed as an investigational drug that could possibly prevent recurrent vertigo in MD. Although phase I and II studies showed promising results, the drug failed to achieve statistical significance for its primary outcome in phase III studies, leading to the cessation of research. Three major phase 3 multicenter randomized clinical trials of OTO-104 were conducted in Europe and the United States. Each of them had definitive vertigo days (DVDs; vertigo attacks lasting at least 20 min, corresponding to a Vertigo Severity Score ≥2) as their primary outcome measure. In study AVERTS-1 (NCT02612337) and the third study (NCT03664674), the difference in DVD between the OTO-104-treated and placebo groups was not significant. Only the European trial AVERTS-2 (NCT02717442), which had the same study design and protocols as AVERTS-1, showed a statistically significant difference. This study, however, was terminated early due to unsatisfactory results of AVERTS-1. Most secondary endpoints were also non-significantly different between the OTO-104 group and placebo in all studies. However, the OTO-104 treatment group showed significantly fewer days sick at home or bedridden at month 3 vs. placebo in AVERTS-2 and NCT03664674 (34).

Although the studies’ outcomes are disappointing, certain findings suggest future research directions. For instance, the placebo response was unusually high in all three studies, and the underlying cause remains unclear. Knowledge of the pathological processes underlying MD is still incomplete. It might be the reason for problems with designing the studies on the medications treating it accurately (34). Currently, efforts should be made to understand the MD pathogenesis better, design trials minimizing the placebo effect, and pursue new drugs that would at least lower the necessity of the destructive treatment of the inner ear.

### SPT-2101

Over the past year, the first reports on the precise intratympanic application of a long-acting steroid formulation containing 6% dexamethasone (SPT-2101) were published (35, 36). In a prospective pilot study, 10 patients with definite MD were enrolled and received a single injection of SPT-2101 into the round window membrane under direct endoscopic visualization. The authors evaluated vertigo control and reported a decrease in the average number of DVDs from 7.6 during the baseline month to 3.3 in the first month, 3.7 in the second month, and 1.9 in the third month. Moreover, 7 out of 10 patients experienced no DVDs during the third month following intratympanic injection of SPT-2101 (36). It is important to note that further studies involving larger population and controlled study designs are required to validate these preliminary observations.

The phase 1b/2a clinical trial of SPT-2101 was completed in Australia in 2024 (ACTRN12621000964819). As of manuscript submission, only preliminary results presented at the 2024 American Academy of Otolaryngology-Head and Neck Surgery Annual Meeting were available, while a full peer-reviewed publication had not yet been released. According to that online report, SPT-2101 demonstrated a favorable safety profile and promising clinical outcomes (37). However, preliminary online reports and conference papers should be interpreted with caution until analyzed and validated results will be published in peer-reviewed full-text publication.

### Intratympanic gentamicin injections

Intratympanic injections may also have vestibuloablative character. Although gentamicin has been available for several decades, recently some studies regarding its role in the management of refractory Ménière’s disease were published.

In a systematic review with meta-analysis, Sharifi et al. (38) compared intratympanic gentamicin and surgical methods. Their analysis confirmed that vestibular neurectomy achieved the highest rate of vertigo. Among intratympanic therapies, gentamicin demonstrated higher vertigo control rate compared with corticosteroids (*p* = 0.001). Similarly, a recent meta-analysis by Wu et al. (39) reported higher vertigo control rates in patients treated with intratympanic gentamicin, however, intratympanic corticosteroids were associated with better pure-tone audiometry outcomes. These findings should be interpreted with caution due to the substantial heterogeneity among the included studies. In a comparative study, Öztürk and Ata (40) demonstrated that intratympanic injections with a mixture of gentamicin and dexamethasone provided significantly better long-term vertigo control than dexamethasone alone after 2 years of follow-up (81.0% vs. 70.6%, *p* = 0.0286), while hearing remained unchanged in 95.2% of treated patients. Despite its efficacy, intratympanic gentamicin should be always considered ototoxic therapy, making careful patient selection essential. The potential long-term consequences of gentamicin therapy should be considered, particularly in patients who may require cochlear implantation in the future. Böscke et al. (41) found significantly poorer post-implantation speech perception outcomes in Ménière’s disease patients previously treated with intratympanic gentamicin (*p* = 0.014).

Nevertheless, intratympanic gentamicin exerts a predominantly vestibulotoxic effect and is intended to achieve selective vestibular ablation while preserving cochlear function whenever possible. Long-term clinical data support this concept. In a retrospective study of 105 patients with definite Ménière’s disease, including nine treated with intratympanic gentamicin, vertigo attacks were significantly reduced after treatment, while hearing outcomes did not differ significantly from the natural course of the disease, suggesting no increased risk of hearing deterioration compared with spontaneous disease progression (42).

## Surgical techniques

**Figure 2:**
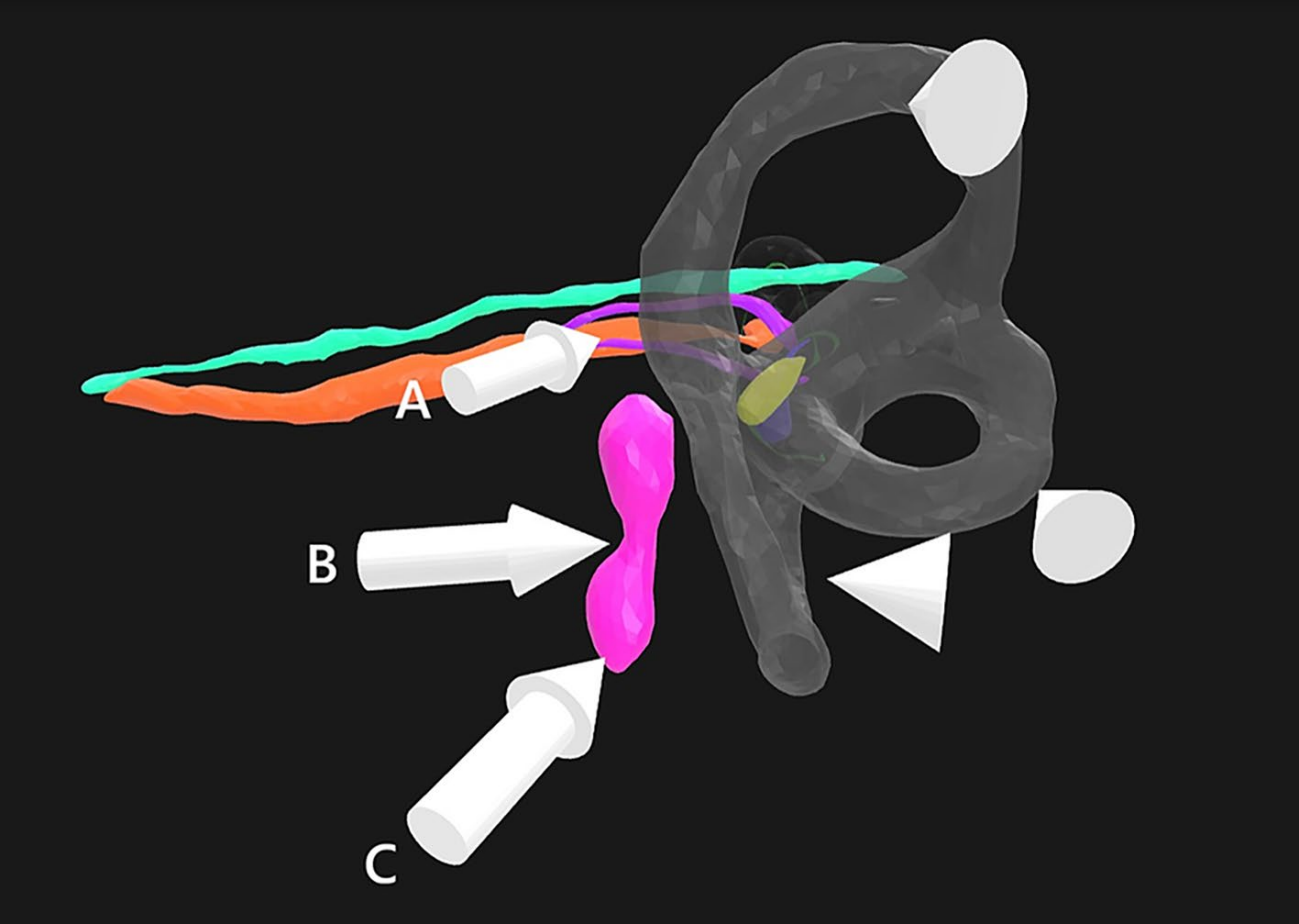
Three-dimensional segmentation of right-sided inner ear structures, including the facial nerve (VII) and vestibulocochlear nerve (VIII), illustrating the key anatomical targets of contemporary surgical procedures for Ménière’s disease. The model was reconstructed from the unaffected ear of a patient with Ménière’s disease using a combination of FIESTA and contrast-enhanced FLAIR sequences acquired 4 h after gadolinium administration. Anatomical landmarks corresponding to sites of established surgical interventions are indicated. Arrow A denotes the vestibular portion of the vestibulocochlear nerve, a target in transcanal vestibular neurectomy. Arrow B indicates the endolymphatic duct, where endolymphatic duct blockage procedures are performed. Arrow C marks the endolymphatic sac, the site of endolymphatic sac surgery. Arrowheads identify the semicircular canals, which represent targets for semicircular canal plugging procedures. The figure provides an anatomical overview of the principal surgical approaches discussed in this review and highlights the relationship between the inner ear structures and their corresponding operative targets.

In about 10%–20% of patients with MD, conservative and intratympanic therapy does not allow for achieving a satisfactory vertigo control and quality of life improvement (1, 3–5). In this group, surgical treatment is indicated. Operative techniques can be categorized into vestibular function–sparing and radical vestibuloablative procedures (Table 2; Figures 1, 2) (1, 3–5, 43).

**Table 2:** Innovations in surgical therapies of Ménière’s disease.

Surgical method	Target anatomical structure	Labyrinth destruction	Hearing preservation potential
Endolymphatic sac decompression	Endolymphatic sac	No	High
Endolymphatic duct blockage	Endolymphatic duct	No	High
Canal plugging (single canal)	Semicircular canal	Partial	Moderate to high
Triple semicircular canal occlusion	All semicircular canals	Partial/complete	Moderate
Vestibular neurectomy	Vestibular nerve	Complete (functional)	Depends on surgical approach^a^

a Hearing preservation potential reflects likelihood of maintaining cochlear function. Labyrinth destruction refers to functional loss of vestibular system.

A recent Cochrane systematic review analyzing surgical treatment of MD highlighted the limited availability of high-quality studies’ evidence (16). Only two randomized controlled trials (total population of 178 patients) met the inclusion criteria: one evaluating tympanic ventilation tubes and the other endolymphatic sac decompression (44, 45). For both methods, the certainty of evidence was rated as low or very low. Moreover, important outcomes such as quality of life, adverse events, and tinnitus were inconsistently reported. The lack of high-certainty evidence from randomized controlled trials highlights the need for adequately powered, well-designed studies and standardization of outcome measures and follow-up protocols. As Cochrane review included only randomized controlled trials, we decided to discuss results of prospective cohort studies and case series describing newer surgical techniques, to highlight the need for larger, well-designed clinical trials. Although preliminary, these studies provide important insights into the pathophysiology of endolymphatic hydrops and potential future treatment strategies.

### Endolymphatic sac surgery

The endolymphatic system consists of vestibular end organs connected to the endolymphatic sac, which serves as a reservoir where the fluid is absorbed into the cerebrospinal fluid (46). In consequence, decompression of the endolymphatic sac would presumably improve endolymph circulation.

Endolymphatic sac surgery, a non-destructive surgical therapy used for patients with Meniere’s disease refractory to medical treatment, is a matter of significant controversy (1, 38). It is broadly divided into four types: endolymphatic sac incision, endolymphatic subarachnoid shunting, endolymphatic mastoid shunting, and endolymphatic decompression (1, 3–5, 36). Recently, studies have described additional benefits of intraoperative steroid use (47, 48).

The procedure, first described in 1927 by Portmann (49), gained popularity in the 1960s. Its efficacy was questioned by a randomized double-blind Danish Sham Surgery Study, which demonstrated that both endolymphatic sac surgery and sham surgery—mastoidectomy groups showed similar significant vertigo control, which is consistent with the strong placebo effect characteristic of Meniere’s disease (45). The AAO-HNS guidelines, published in 2020, make no recommendation on endolymphatic sac decompression due to its uncertain benefit and insufficient quality of evidence (1). However critical review of the reports pertaining the efficacy of endolymphatic sac surgery allowed the following conclusions to be made: (1) approximately 80%–90% of patients have total or substantial vertigo control at 2 years after surgery, with an increasing period of follow-up, the results deteriorate; (2) results of the various surgical modifications to endolymphatic sac surgery are essentially equivalent; and (3) risk of complications is low, with <2% incidence of complete SNHL (1). Lee et al. (16) in the Cochrane Database of Systematic Reviews were also unable to draw meaningful conclusions about the efficacy of endolymphatic sac surgery for Ménière’s disease. The mechanism of sac surgery for vertigo control remains unclear. Results of imaging analysis show that endolymphatic hydrops is not significantly reduced by endolymphatic sac surgery, suggesting that the mechanism by which it controls vertigo is unrelated to hydrops improvement (50).

Recently, several publications described results of endolymphatic sac surgery (51–53). A systematic literature review and meta-analysis showed that surgery leads to significant functional improvement and relief of vertigo; however, it is associated with a reduction in auditory abilities (average hearing loss of 9.25 dB and a 26.23% deterioration in speech comprehension after surgery) (51). Gendre et al. (52) observed that endolymphatic sac surgery does not negatively affect vestibular function.

A modification of the procedure involving intraendolymphatic sac steroid administration was proposed by Kitahara et al. (54). After the decompression and drainage, the endolymphatic sac is filled with 20 mg of prednisolone. Then, a bundle of five absorbable gelatin films impregnated with high-concentration dexamethasone (32 mg/4 mL) is inserted into the sac lumen, allowing gradual delivery of dexamethasone into the sac after surgery. Authors reported the results of a randomized controlled study comparing long-term outcomes in patients after classical endolymphatic sac drainage with those treated with a modified technique that included intraoperative steroid administration. Total vertigo control was achieved in 85.1% and 88% of patients, respectively. Despite satisfactory results, no statistical difference was found between these groups. Still, postoperative audiological results were significantly better in patients with intraoperative steroid administration.

The authors of the International consensus (ICON) on treatment of Ménière’s disease agree that ESS should be the first option after failure of the medical conservative treatment, if hearing function is useful (5).

The surgical failure may be due to intraoperative difficulty in identifying the endolymphatic sac. Moreover, obstruction of the endolymphatic space may occur proximally to the sac. Recently, Salvinelli et al. (53) proposed a modification of the sac decompression extended to duct decompression. The surgery is performed via an antromastoidectomy approach similar to classic endolymphatic sac surgery. In this technique, precise skeletonization of the dura mater, extending to the posterior semicircular canal arch, is necessary to localize and decompress the duct. In a study evaluating 14-month follow-up in 82 patients, the authors analyzed changes in the DHI questionnaire, subjective severity of vestibular and aural symptoms, and pure-tone audiometry results. After the surgery, vertigo control was achieved in 89% of patients. Eight percent of the group (seven patients) underwent subsequent ablative treatment (vestibular neurectomy/intratympanic gentamicin), while the remaining 3% (two patients) refused further treatment escalation. Statistically significant improvement of the DHI results was reported—average DHI scores were 70 and 18, before and after the surgery, respectively (*p* < 0.001). At the follow-up visit, aural fullness decreased in 43% of patients and completely resolved in 40%. In tinnitus analysis, partial and complete improvement were observed in 38% and 22% of cases, respectively. Moreover, no hearing deterioration was reported in any of the patients.

### Endolymphatic duct blockage

In 2015, Saliba et al. (55) introduced a novel surgical technique on the endolymphatic system, founded on a paradigm shift in the pathophysiological understanding of MD. Their theory originated from descriptions of numerous dark cells producing endolymph in the endolymphatic sac (43). Moreover, surprisingly, endolymphatic duct obliteration in patients with acoustic neuroma operated via retrolabyrinhtine approach does not lead to endolymphatic hydrops (56, 57). In consequence, Saliba et al. (55) proposed that dysfunction of the endolymphatic sac could underlie endolymphatic hydrops.

Endolymphatic duct blockage surgery begins with mastoidectomy and endolymphatic sac decompression as described above. Then, identification and skeletonization of the endolymphatic duct in its superior and inferior parts are performed. The endolymphatic duct is then blocked with two small titanium clips.

A randomized controlled trial comparing the results of the new procedure with those of endolymphatic sac decompression was conducted. At a 2-year follow-up, vertigo control was significantly better in the group treated with the new surgical technique: complete attack reduction was achieved in 96.5% of patients, compared to 37.5% after classic endolymphatic sac decompression (*p* = 0.002). Hearing outcomes were satisfactory, with hearing preserved in both groups and no significant difference between pre- and postoperative results (49). What is more, Gabra et al. (58) reported improvement in quality of life in patients with MD after endolymphatic duct blockage. In a study of 54 patients treated with endolymphatic duct blockage, the mean quality-of-life score increased from 21.4 preoperatively to 64.6 after surgery (*p* < 0.001). In addition, 89.9% of patients remained free of Ménière’s attacks during follow-up. Nevertheless, the potential contribution of placebo effects and the long-term durability of symptom control remain incompletely understood.

### Semicircular canal plugging

The concept of semicircular canal occlusion was first proposed in 1990 by Parnes and McClure (59) in the context of BPPV management. Then, in 1998, Minor et al. (60) introduced clinical results of canal plugging in patients with superior semicircular canal dehiscence. Subsequently, numerous studies expanding the therapeutic potential of canal-targeted interventions in patients with severe MD were published (61–67). The main advantage of these methods is the preservation of the remaining vestibular organs’ function and hearing. The surgical concept varies, ranging from a single lateral or posterior semicircular canal occlusion to triple-canal plugging, with optional additional vestibular obliteration.

Results of lateral semicircular canal plugging in 28 patients with MD were evaluated by Charpiot et al. (66). After the surgery, canal paresis assessed in caloric tests was still evident in all patients. Two years after canal plugging, 16 patients were available for follow-up evaluation. Complete or substantial vertigo control was achieved in 75% of these patients, including complete restoration of normal daily activities (AAO-HNS class A) in 62.5% and no functional limitation (class B) in 12.5%. Postoperative hearing preservation was achieved in 82% of cases. Despite these promising results, findings should be interpreted with caution given the small sample size and uncontrolled study design.

Posterior semicircular canal occlusion was proposed as a surgical treatment in nine patients with Tumarkin’s drop attacks (63). Postoperative vHIT examination showed an isolated significant decrease in posterior canal gain median (0.52, compared to 0.86 before surgery; *p* < 0.009) with no changes in anterior or lateral canals. All nine patients reported complete resolution of Tumarkin’s attacks. After surgery, hearing deterioration of >10 dB in the PTA was observed in two patients, with a mean PTA change of 9.45 dB.

Triple semicircular canal plugging was reviewed in a meta-analysis by Zhu et al. (61). The authors included seven studies reporting postoperative audiovestibular results in a cohort of 367 patients with MD. They concluded that triple canal occlusion is an effective treatment allowing for complete or substantial vertigo control (class A/B according to AAO-HNS) in 99% of patients. The surgery was associated with a 22% incidence of hearing loss with a PTA level decline of approximately 10 dB–20 dB. Jiang et al. (67) compared the results of 36 patients with MD treated surgically with vestibular neurectomy, triple canal occlusion. Still, only 10 patients treated with each procedure completed follow-up. Authors concluded that both methods had similar efficacy for vertigo attack control (*p* = 0.839), with the majority of patients obtaining vertigo control class A according to AAO-HNS (1). Moreover, postoperative unsteadiness was observed in 57% of patients in the neurectomy group, whereas none in the triple canal plugging group reported persistent postoperative balance problems (*p* = 0.025). Nevertheless, further studies are necessary as only 10 patients treated with canal occlusion were described in that article.

### Transcanal neurectomy

After previous therapy failure, in patients with severe Meniere’s episodes, especially Tumarkin’ drop attacks, ablative surgery including vestibular neurectomy and labyrinthectomy can be indicated (1–5).

Classical surgical techniques for vestibular nerve section are retrosigmoidal and middle fossa approaches (68, 69). Another option for surgical management of Meniere’s disease is the endoscopic approach through the external acoustic meatus and the tympanic cavity. There are two options for this corridor—infracochlear and transvestibular (70, 71). It should be noted that transmeatal techniques remain experimental, with evidence limited to cadaveric studies and isolated case reports. These approaches enable transmeatal access, thereby decreasing both operative time and post-operative morbidity. The use of skull base navigation may also enhance patient safety regarding vital neurovascular structures, including the facial nerve, cochlea, jugular bulb, and carotid artery. In both infracochlear and transvestibular approaches, curved 2- and 3-mm diamond burrs are used, so the dimensions of the surgical tunnel are minuscule compared to those of a craniotomy. A small diameter of the approach may decrease the risk of post-operative cerebrospinal fluid leak.

Both approaches begin with the use of a 0-degree endoscope and the elevation of the tympanomeatal flap. The flap is elevated, preserving the integrity of the ossicular chain. Access is made to the hypotympanum through the lower quadrants of the raised tympanic membrane.

The infracochlear transcanal approach remains an experimental surgical technique, with the currently available evidence limited to cadaveric studies. To proceed via the infracochlear route, the bone over the cochlea is thinned, and hypotympanotomy is performed, which serves as the superior limitation of the approach. Then, the carotid artery is identified in its canal anteriorly, and the jugular bulb in its fossa postero-inferiorly. Once the tunnel passes inferiorly to the basal turn of the cochlea, a modified, curved curette is used to remove the bone superiorly and enter the internal acoustic meatus. Then, with the use of 30-degree endoscope, the inferior vestibular nerve is identified and cut with the sharp hook. After that, the superior vestibular nerve is cut in the same manner. The small fat graft is then placed in the tunnel like a plug, the tympanomeatal flap is restored, and the procedure is finished. In this approach, the hearing may be preserved. Nevertheless, to date, the infracochlear endoscopic approach has been described in cadaveric study only (71).

The transmeatal transpromontorial approach described by Marchioni et al. (70) was reported in two clinical cases and requires a more extensive surgical tunnel. After the tympanomeatal flap is raised, the tympanic membrane is dissected from the malleus in order to expose the medial wall of the tympanic cavity fully. The incus is disarticulated, and the stapes is removed from the oval window in order to visualize the vestibule and spherical recess. Then, the oval window opening is widened with the curette. The fundus of the internal acoustic meatus is then opened by curetting the spherical recess. The inferior vestibular neurotomy is then made in this area by detaching the nerve from the spherical recess. After that, the postero-superiorly located elliptical recess is dissected with the curette, and the superior vestibular nerve is cut. To prevent post-operative CSF leaks, multilayer reconstruction should be performed. The fat graft serves as the first layer; then, the vestibule is filled with muscle, and fibrin glue is placed over the two layers. Then, the adipose tissue covers the area of the oval window. The tympanomeatal flap is repositioned, and gelfoam pieces are placed over the tympanic membrane to ensure it is stable. This approach may be used in the selected patients with non-serviceable hearing. Authors described two patients with disabling MD treated with this method and reported complete vertigo remission in 6-months follow-up. Given the very limited clinical experience, larger prospective studies are necessary to validate these preliminary findings.

## Study limitations

Several limitations of this review should be acknowledged. First, narrative character of the literature review can cause selection bias as the process of study identification and inclusion was not conducted according to a predefined systematic review protocol. The aim of the present article was to provide a comprehensive overview of innovative pharmacological and surgical therapies for Ménière’s disease, including investigational methods. For some of them only preliminary data are currently available. Thus, a systematic review methodology cannot be made properly at this time. Second, many of the innovative therapeutic approaches discussed are supported by limited clinical evidence, including small patient cohorts, early-phase clinical trials, conference abstracts, online preliminary reports through company communications rather than peer-reviewed publications. Consequently, the findings presented should be interpreted with caution.

Moreover, most studies included small patient cohorts and relatively short follow-up periods. Furthermore, studies discussed in the review are heterogenous in terms of patient selection criteria, treatment protocols, outcome measures, and duration of follow-up, which limits direct comparisons between interventions. Consequently, additional high-quality studies are required to validate the reported outcomes. Finally, the fluctuating course of Ménière’s disease and the potential influence of placebo effects complicate the assessment of treatment efficacy.

In the present review, we aimed to provide an overview of novel therapeutic concepts that may represent promising directions for future research. Several of the interventions discussed are of particular interest because they not only offer potential new treatment strategies but also contribute to the ongoing discussion regarding the pathophysiology of Ménière’s disease. Thus, some of therapies discussed should be interpreted as potential directions for future research rather than established standards of care.

## Conclusion

Ménière’s disease is characterized by high interindividual variability and unpredictable clinical course, making it not only diagnostic but also therapeutic challenge. This clinical heterogeneity suggests that no single treatment strategy is likely to be effective for all patients, highlighting the need for a personalized therapeutic approach. Nowadays, research focus on identifying interventions reducing vertigo attacks with hearing preservation, thus, growing attention has been directed to otoprotective drugs. In consequence, recent advances in pharmacological therapy include novel agents targeting oxidative stress, immune-mediated pathways, and neuromodulatory processes involved in vestibular function. Still, modifications of betahistine aimed at improving its bioavailability are also under investigation. Among pharmacological therapies, SPI-1005 (ebselen) currently has the most advanced clinical evidence and has shown promising results in phase 3 studies, although the complete peer-reviewed data are still awaited. Rimegepant may represent a potential therapeutic option in patients with Ménière’s disease and migraine, although larger controlled studies are required. Analyzing intratympanic therapies, such as OTO-104, no superiority over placebo was documented in randomized clinical trials. Moreover, innovative surgical methods aimed to partially or completely preserve vestibular function are proposed as a trend toward selective, patient-tailored surgical management. Endolymphatic duct blockage and canal plugging may demonstrate vertigo control while offering the potential to preserve auditory and vestibular function in selected patients. Nevertheless, further studies on large population with adequate end-points are needed to confirm long-term efficacy of novel treatment methods and redefine therapeutic algorithm.
